# Hypercholesterolemia and a candidate gene within the 12q24 locus

**DOI:** 10.1186/1475-2840-10-38

**Published:** 2011-05-09

**Authors:** Claudia Gragnoli

**Affiliations:** 1 Laboratory of Molecular Genetics of Complex and Monogenic Disorders, Department of Medicine and Cellular & Molecular Physiology and Biostatistics, Penn State University and M. S. Hershey Medical Center, Hershey, PA, USA; 2 Center for Biotechnology and Department of Biology, Temple University's College of Science & Technology, Philadelphia, PA, USA; 3 Molecular Biology Laboratory, Bios Biotech Multi-Diagnostic Health Center, Rome, Italy

**Keywords:** SNPs, *PSMD9*, Linkage, Hypercholesterolemia

## Abstract

**Background:**

The 12q24 locus entails at least one gene responsible for hypercholesterolemia. Within the 12q24 locus lies the gene of proteasome modulator 9 (*PSMD9*). *PSMD9 *is in linkage with type 2 diabetes (T2D), T2D-nephropathy and macrovascular pathology in Italian families and *PSMD9 *rare mutations contribute to T2D.

**Aims:**

In the present study, we aimed at determining whether the *PSMD9 *T2D risk single nucleotide polymorphisms (SNPs) *IVS3 + nt460 A > G, IVS3 + nt437 T > C *and *E197G A > G *are linked to hypercholesterolemia in 200 T2D Italian families.

**Methods:**

We characterized 200 Italian families for presence and/or absence of hypercholesterolemia characterized by LDL levels ≥ 100 mg/dl in drug-naïve patients and/or presence of a diagnosis of hypercholesterolemia in a patient treated with statin medication. The phenotypes were described as unknown in all cases in which the diagnosis was either unclear or the data were missing. We tested in the 200 Italians families for evidence of linkage of the *PSMD9 *SNPs with hypercholesterolemia. The non-parametric linkage analysis was performed for the qualitative phenotype by using the Merlin software; the Lod score and correspondent P-value were calculated. For the significant linkage score, 1000 replicates were performed to calculate the empirical P-value.

**Results:**

The *PSMD9 *gene SNPs studied show linkage to hypercholesterolemia. The results are not due to random chance.

**Conclusions:**

*PSMD9 *should be tested in all populations reporting linkage to hypercholesterolemia within the chromosome 12q24 locus. The impact of this gene on hypercholesterolemia and contribution to cardio- and cerebrovascular events may be high.

## Introduction

The chromosome 12q24 locus is linked to dyslipidemia and hypercholesterolemia and must entail at least one gene responsible for these phenotypes [1,2]. Dyslipidemia is an important cardiovascular risk factor for T2D. Dyslipidemia is a major modifiable risk factor of atherosclerosis, which introduces the possibility of both treatment and prevention of the cardiovascular complications of T2D [3].

Within the 12q24 locus is located the gene of proteasome modulator 9 (*PSMD9*). PSMD 9 is highly expressed in pancreatic islets and it is a coactivator of insulin gene transcription, and *PSMD9 *gene dosage abnormalities cause beta-cell dysfunction and contribute to type 2 diabetes (T2D) [4]. PSMD9 is a ubiquitously expressed protein. We reported that *PSMD9 *rarely causes T2D by unique mutations [5] and that the *PSMD9 IVS3 + nt460 A > G, IVS3 + nt437 C > T *and *E197G A > G *single nucleotide polymorphisms (SNPs) are linked to late-onset T2D via the recessive model [6] and to MODY3 [7] via the additive model. We recently published that the *PSMD9 IVS3 + nt460 A > G, IVS3 + nt437 C > T *and *E197G A > G *T2D risk SNPs are linked to T2D-nephropathy [8] as well as to macrovascular pathology [9]. Insulin is a lipid-synthetic hormone, thus alteration in a gene regulating insulin gene transcription may alter lipid metabolism as well and contribute to hypercholesterolemia. Furthermore, overexpression in transgenic mice of the coactivator Bridge-1, homologous of PSMD9, results in elevated triglyceride levels in Bridge-1/PSMD9 with severe diabetes compared to non-transgenic control mice [10].

We aimed at identifying in the present study whether the coactivator of insulin transcription *PSMD9 *may contribute to linkage to hypercholesterolemia in 200 Italian T2D families. Thus, we tested the *PSMD9 *T2D risk SNPs for linkage to hypercholesterolemia.

## Methods

### Ethics Statement

the subjects were all recruited following the Helsinki declaration guidelines. Subjects gave written informed consent and the Penn State College of Medicine Ethical Committee approved the study.

### Families

We recruited 200 Italian T2D affected siblings and families. We excluded families with identical twins and we confirmed that our families were at least for three generations Italians and had no known ancestor of not-Italian origin. The Italian population lives in a peninsula and it is very suitable for genetic studies as there are no concerns for genetic admixture.

### Clinical Phenotyping

We characterized the Italian family's subjects, including those unaffected by T2D, for LDL cholesterol ≥100 mg/dl in drug-naïve patients and/or presence of hypercholesterolemia in patients treated with statin medication.

Phenotypes are described as unknown if diagnosis is unclear or data are missing. In our 200 Italian families, hypercholesterolemia is present in 87.30% of the families.

### Sequencing

We amplified by PCR the *IVS3 PSMD9 *region containing the *+nt460 A > G *and *+nt437 C > T *SNPs and the exon 5 coding region containing the *E197G A > G *SNP with specific primers [5] in the affected and unaffected family members. We directly sequenced the PCR products, status post-purification via EXOSAP-IT on an automated ABI 3730 Sequencer.

### Statistical Analysis

We tested in the 200 Italians families for linkage of the *PSMD9 *above-mentioned SNPs with hypercholesterolemia. Non-parametric linkage analysis for the qualitative phenotype was performed for the three SNPs via the latest version of Merlin software [11]. Merlin analyzed all the informative families (n = 84) within this dataset. The number of individuals in the 200 families is 928; the number of individuals in the 84 families is 390 (table 1).

**Table 1:** Population Demographics

Population	T2D Families/Subjects	Sex (F/M)	Families Informative for Hypercholesterolemia/Subjects	Sex (F/M)
**100% Italians**	200/928	467/461	84/390	199/191

F = female, M = male.

Allele frequencies were calculated from the data. The result is reported in LOD score as calculated by Merlin.

To rule out the presence of a false positive in our result, we calculated how many times P-values equal or inferior to the real one were expected by chance in 1,000 replicates of simulations by using the gene dropping method: this analysis replaces real data with simulated data, while maintaining the pedigree structure, allele frequencies and recombination fraction. These datasets are generated under the null hypothesis of no linkage.

## Results

The results of the non-parametric linkage analysis performed are reported in table 2 with the LOD score, the corresponding P-value and the empirical P-value. The tested *PSMD9 *SNPs are significantly linked to hypercholesterolemia in the T2D-families. The three variants tested contribute almost equally to the results, with a non significant prevalence of the intronic variants over the coding variant.

**Table 2:** Non-parametric Linkage Analysis of Hypercholesterolemia of the Italian Families by Merlin software

Phenotype	Prevalence	Families	Z Score	Lod	P	Empirical P
**Hypercholesterolemia**	87.30%	84	2.12	1.24	0.008	0.007

Prevalence = phenotype prevalence among the family subjects studied; Families = families number analyzed; Z score = derived from the non-parametric linkage analysis by Merlin; Lod = Lod score derived from the non-parametric linkage analysis by Merlin; P = p-value; Empirical P = p-value derived from 1000 replicates by using the gene dropping method; Hypercholesterolemia defined by the presence of LDL ≥ 100 mg/dl in drug-naïve patients or by the presence of a diagnosis of hypercholesterolemia treated with a statin medication.

## Discussion

Our study shows that the *PSMD9 *SNPs studied are in linkage with hypercholesterolemia in the Italian T2D families, thus making *PSMD9 *a possible risk gene for hypercholesterolemia. However, these findings should be replicated in families with hypercholesterolemia of other ethnicities and in particular in all ethnical groups in which the chromosome 12q24 locus was reported as linked to elevated cholesterol [1,2] to confirm the general relevance of the gene in other populations as well.

The finding of our study may have an inestimable impact given the relevance of the 12q24 locus in hypercholesterolemia in several ethnical groups [1,2] as well as the importance of hypercholesterolemia as a major risk factor for macrovascular disease and cerebral and cardiovascular events reducing quality of life and lifetime span [3]. Given the *PSMD9 *linkage reported with T2D-nephropathy [8] and with macrovascular pathology [9] as well, it is possible that the PSMD9 has pleiotropic effects contributing to the phenotypes associated with macrovascular pathology as well as with T2D. PSMD9 is present at high concentrations in eukaryotic cells and is part of the 26S proteasome complex, which contributes to the degradation of intracellular proteins in antigenic peptides in the immune response to antigen presentation by MHC class I cells (http://www.genecards.org/cgi-bin/carddisp.pl?gene=PSMD9). Thus, one potential role of PSMD9 in the phenotypes associated with T2D and atherosclerosis could be related to a direct role in the pathogenesis of inflammation as an autoimmune process [12]. In addition, PSMD9 is mediating the magnitude and duration of the transcription of the ligand-dependent retinoid-target genes (http://www.millipore.com/pathways/pathviewer.do?pathwayId=76). Thus, PSMD9 may, if impaired in its function due to gene variants affecting the protein sequence and/or protein dosage, alter dosage and effects of several downstream genes. In transgenic mice with pancreatic overexpression of the homologous of PSMD9, it has been shown that the increased dose of the PSMD9 protein is causing insulin deficiency and diabetes as well as hypertriglyceridemia [10]. In addition, an inhibition of the gene transcription in vitro also reduces insulin secretion [13]. Thus, the hypothesis is that given the complex network of transcription and co-activator factors that PSMD9 is associated to, both PSMD9 reduced or increased protein dose in the cell may have pathogenetic effects, by potentially contributing to phenotypes that may be either different or having an underlying contributing factor.

## Conclusions

We conclude that the *PSMD9 *SNPs *IVS3 + nt460 A > G, IVS3 + nt437 C > T *and *E197G A > G *are linked to the hypercholesterolemia phenotype in the Italian T2D-families tested. This finding is of relevance as hypercholesterolemia is a major contributor of cardiovascular disease in T2D, and thus the identification of genes implicated in the pathogenesis of hypercholesterolemia may lead to future primary and secondary prevention therapies implementations.

## Competing interests

The author declares that they have no competing interests.

## Authors' contributions

CG conceived and designed the study, collected the clinical information, performed the statistical analysis and drafted the manuscript.
